# Reconstruction of Craniomaxillofacial Bone Defects with 3D-Printed Bioceramic Implants: Scoping Review and Clinical Case Series

**DOI:** 10.3390/jcm13102805

**Published:** 2024-05-09

**Authors:** Maarten Verbist, Anne-Laure Vandevelde, Joris Geusens, Yi Sun, Eman Shaheen, Robin Willaert

**Affiliations:** 1 Department of Oral and Maxillofacial Surgery, University Hospitals Leuven, 3000 Leuven, Belgium; 2 OMFS IMPATH Research Group: Department of Oral and Maxillofacial Surgery, Imaging and Pathology, Leuven University Hospitals, 3000 Leuven, Belgium

**Keywords:** plastic surgery, reconstructive surgical procedures, patient-specific implants, bioceramics, hydroxyapatite, three-dimensional printing, bone grafts

## Abstract

Reconstruction of craniomaxillofacial bone defects using 3D-printed hydroxyapatite (HA) bioceramic patient-specific implants (PSIs) is a new technique with great potential. This study aimed to investigate the advantages, disadvantages, and clinical outcomes of these implants in craniomaxillofacial surgeries. The PubMed and Embase databases were searched for patients with craniomaxillofacial bone defects treated with bioceramic PSIs. Clinical outcomes such as biocompatibility, biomechanical properties, and aesthetics were evaluated and compared to those of commonly used titanium or poly-ether-ether-ketone (PEEK) implants and autologous bone grafts. Two clinical cases are presented to illustrate the surgical procedure and clinical outcomes of HA bioceramic PSIs. Literature review showed better a biocompatibility of HA PSIs than titanium and PEEK. The initial biomechanical properties were inferior to those of autologous bone grafts, PEEK, and titanium but improved when integrated. Satisfactory aesthetic results were found in our two clinical cases with good stability and absence of bone resorption or infection. Radiological signs of osteogenesis were observed in the two clinical cases six months postoperatively. HA bioceramic PSIs have excellent biocompatible properties and imitate natural bone biomechanically and radiologically. They are a well-suited alternative for conventional biomaterials in the reconstruction of load-sharing bone defects in the craniomaxillofacial region.

## 1. Introduction

Bone defects in the craniomaxillofacial region can cause significant aesthetic and functional problems, resulting in a decreased quality of life (QoL) [1]. The etiology of these defects can be derived from trauma, infection, oncologic surgery, or congenital disorders [1,2]. Reconstructive surgery is important for restoring the aesthetic and functional role of these complex anatomical regions and can be challenging for both patients and surgeons [1]. In previous cases described in the literature, autologous bone grafting, titanium, or poly-ether-ether-ketone (PEEK) implants have been used to cover bone defects [3]. However, each material exhibits certain limitations, raising the need for further research to determine the best application for bone reconstruction in the craniomaxillofacial region. General requirements for an ideal implant are biocompatibility, non-allergenic behavior, radiopacity, cost-effectiveness, ease of use, and adequate strength [1,4,5]. In addition, implants used for osseous reconstruction are even more favorable if they possess important biological properties such as osteoconduction and osteoinduction to enhance implant ingrowth and dimensional stability [1,6,7]. Osteoconduction is defined as the ability to passively host bone-generating cells, such as osteoblasts, and guide their migration into the graft to enhance its ingrowth [2,8]. A microscopic, porous structure is necessary to allow migration into a three-dimensional structure [9]. Osteoinduction, on the other hand, refers to the capacity to recruit and stimulate the proliferation and differentiation of pluripotent mesenchymal cells into osteoblasts. Osteogenesis is the ability to contain progenitor cells, growth factors, and a matrix to form new bone [2,8]. Factors such as bone morphogenetic protein (BMP), fibroblast growth factor (FGF), and vascular endothelial growth factor (VEGF) are necessary to induce new bone formation [2]. An ideal bone substitute has the aforementioned properties and accomplishes osteointegration. This can be defined as the ingrowth of an implant due to the formation of bone tissue at the bone–implant interface in the absence of fibrous tissue formation [2,8]. It is difficult to mimic the dynamic properties of natural bone with those of conventional implants. A mineral matrix is necessary to provide stability for osteoblasts, osteoclasts, and osteocytes, as well as growth factors and adequate vascularity.

Autologous bone grafting has the best biocompatibility because it possesses osteoconductive, osteoinductive, and osteogenic properties and is therefore considered the gold standard [2,6,8,9,10,11]. Donor site morbidity, limited availability of suitable donor bone, acceptor site resorption, and longer surgery time are major drawbacks [6,10,11]. Titanium is a popular material owing to its biocompatibility, osseointegration capacity, and strength [5,12]. However, it causes radiological artefacts, thermal discomfort, and has a higher infection rate than bioceramics and bone grafts [9,13,14]. PEEK implants exhibit good strength, poor bioactivity, and poor osteoconductive properties [9,12,15]. Recently, bioceramic patient-specific implants (PSIs) have gained attention, providing a valuable alternative to conventional materials for the reconstruction of bone defects in the craniomaxillofacial region [6,12,16].

Technological advancements in three-dimensional (3D) printing have the potential to integrate the biomechanical properties of bioceramics in a PSI [12,17,18]. This technique is relatively new but is considered the future of transplant medicine [19]. Computer-aided design and manufacturing (CAD/CAM) of bioceramic PSIs provides the possibility of printing a biocompatible patient-specific scaffold to guide osteoblasts and therefore replace bone defects in the craniomaxillofacial region without donor site morbidity [20]. The implant promotes regeneration and stimulates osteogenesis and fibrovascular ingrowth [18,21]. Hydroxyapatite (HA) is mostly used, sometimes combined with growth factors such as bone morphogenic protein 2 (BMP2) [22,23]. BMP2 functions as an osteoinductive factor enhancing both osteoblast differentiation and angiogenesis. Its application demands a combination of osteoconductive carriers such as autologous and allogenous bone grafts or HA scaffolds [2].

Few studies describe the clinical use of HA bioceramic PSIs in the craniomaxillofacial region [9,16,17]. Moreover, there is a lack of consensus on the optimal balance between strength and osseointegration [16,20]. Mechanical properties differ mainly in pore configuration, which is necessary to enhance bone ingrowth in the implant [24]. For example, the triply periodic minimal surface method (TPMS) has better strength than the conventional pore configuration [20,25]. TPMS was also applied in the PSIs designed for the clinical cases included in this review [22,23].

The objective of this scoping review was to evaluate the use of HA bioceramic PSIs in comparison to titanium, PEEK, and autologous bone grafts in terms of biocompatibility and biomechanical behaviour. Two clinical cases were added to demonstrate the clinical outcomes of these implants in reconstructive surgery of craniomaxillofacial bone defects.

## 2. Materials and Methods

In this scoping review, bioceramic PSIs were compared to conventional techniques to restore bone defects in the craniomaxillofacial region. The included studies were selected using PICOS criteria (patient population, intervention, comparison, outcome, and study design). The inclusion criteria were clinical and preclinical studies that used conventional materials or 3D-printed bioceramic implants. In addition, in vitro studies describing the osseointegration process and biomechanical properties of the selected bioceramics and other bone substitutes were included.

The PRISMA guidelines (Preferred Reporting Items for Systematic Reviews and Meta-Analyses) for scoping reviews were followed, using the PRISMA-ScR Checklist.

### 2.1. Search Strategy

A selective search of digital databases, including PubMed and Embase, was conducted in February 2024. Search terms included “bioceramics”, “hydroxyapatite”, “patient-specific implant”, “implant”, “3D printing”, “craniofacial surgery”, and “craniomaxillofacial bone defects”. Only full-text English articles (publication years 1986 to 2024) that met the inclusion criteria were included in this review. A systematic search was beyond the scope of this study.

### 2.2. Case Presentation

Two patients were treated at University Hospitals Leuven (Leuven, Belgium) with HA bioceramic PSIs. These designs were verified and manufactured by CERHUM (Liège, Belgium). An overview of the sex, etiology, location, surgical approach, bone defect, complications, aesthetic outcome, and follow-up time is presented in Table 1.

### 2.3. Intraosseous Hemangioma—Case 1

**Figure 1:**
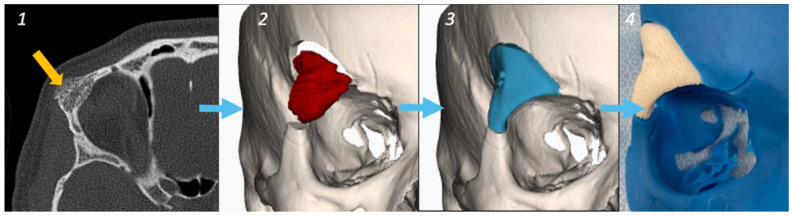
Planning, case 1. (**1**): Axial plane CT image of the supra-orbital region showing an extensive intra-osseous lesion (yellow arrow). (**2**): Digital segmentation of the lesion and planning of the resection margins. (**3**): Virtual design of the dimensions of the PSI. (**4**): Manufacturing and delivery of the implant.

**Figure 2:**
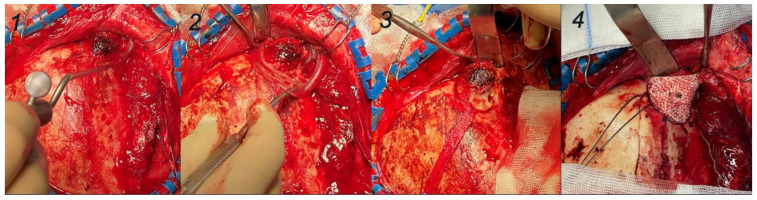
Surgical steps, case 1. (**1**): Decollation of pericranium and visualization of the intraossous hemangioma, and navigation with Brainlab surgical navigation system. (**2**): Fitting of the cutting guide and marking the trepanation line. (**3**): Extraction of the intraosseous haemangioma. (**4**). Insertion of the PSI and fixation with a 15 mm screw (2.0) inside the temporal bone.

A 42-year-old woman was referred with a progressive hard nodule in the right supra-orbital rim, present for five years. There was a bony swelling at the zygomatic process of the frontal bone, without signs of inflammation or infection. Computed Tomography (CT), a Positron Emission Tomography (PET)-CT scan, and a biopsy sample confirmed the diagnosis of an intraosseous hemangioma (Figure 1). An HA bioceramic PSI (MyBone^®^) was designed by an in-house clinical engineer using the exported DICOM (Digital Imaging and Communication in Medicine) files of a high-quality CT scan (0.6 mm slices). Corresponding cutting guides were planned digitally with Mimics and Proplan CMF™ (Materialise, Leuven, Belgium) based on predefined resection margins. The PSI was externally manufactured by CERHUM SA (Liège, Belgium). The porosities were virtually planned and additively manufactured via stereolithography. The dimensional properties were verified using 3D scanning (GOM; ATOS, Braunschweig, Germany). After production, the dimensions and direction of the screw holes were studied on a prototype model provided by the company (Figure 1 (4)) and steam-sterilized inside the hospital (134 °C, 18 min). The well-prepared surgery was then performed in cooperation with the neurosurgical department because of the involvement of the lesion in the frontal bone and its relationship with the dura mater. A hemicoronal incision was made on the right to expose the lesion. Surgical navigation (Brainlab, Munich, Germany) was used to check safe cutting margins (Figure 2). The PSI was first tested in the intended position and was adjusted minimally for perfect placement. An immediate inflow of blood was observed intraoperatively. The implant was fixed using one 15 mm screw and covered with the temporal muscle.

### 2.4. Hemifacial Asymmetry—Case 2

**Figure 3:**
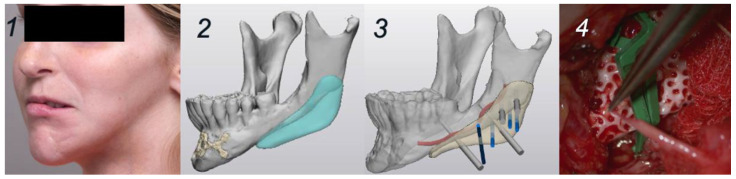
Planning and surgery, case 2. (**1**): Clinical situation pre-surgery, 45° left. (**2**): Digital design of the PSI. (**3**): Digital design of the fixation screws in relation to the alveolar nerve. (**4**). PSI placement and fixation.

A 19-year-old woman was planned for reconstructive surgery of the left mandibular angle as part of a multistage treatment plan for left hemifacial asymmetry secondary to Parry–Romberg syndrome. The HA bioceramic PSI and corresponding cutting guide were virtually planned in Mimics and Proplan CMF™ (Materialise, Leuven, Belgium) mirroring the contralateral side. Based on the 3D CT images (0.6 mm slices), the screw holes were positioned in favorable relation with the alveolar nerve (Figure 3). The manufacturing process was equivalent to the first case. Lipofilling with peri-umbilical fat and a Superficial Circumflex Iliac Artery Perforator (SCIAP) flap were planned for soft tissue reconstruction. The prepared surgery was initiated with an extra-oral approach incising in the peri-angular region on the left side, dissecting and containing important nervous structures such as the marginal branch of the facial nerve. The 3D-printed cutting guide was inserted to drill the screw holes. The PSI was evaluated in the planned position before fixation. After insertion and fixation of the implant using 2 screws, the SCIAP-flap was harvested and anastomosed on the facial artery and vein (Figure 3). Surgery was completed with lipofilling of the left upper lip with harvested free abdominal fat.

## 3. Results

**Figure 4:**
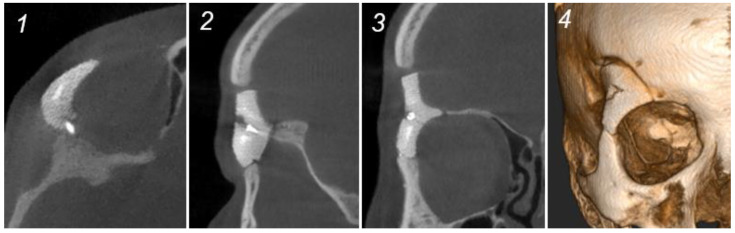
Radiological (CBCT) and clinical situation after 6 months, case 1. (**1**): Axial plane. (**2**): Sagittal plane—signs of osteogenesis. (**3**): Frontal plane—signs of osteogenesis. (**4**): 3D CT image.

The first clinical case showed good ocular vision, normal ocular mobility, and satisfactory aesthetic results with maximal preservation of facial symmetry after one week, two months, and six months of clinical and radiological follow-up (Figure 4). Mild right-sided hypoesthesia of the first and second branches of the trigeminal nerve was reported after two months, which resolved spontaneously six months postoperatively. Good positioning and contouring of the PSI after six months were identified on cone-beam CT (CBCT) imaging (Figure 4).

**Figure 5:**
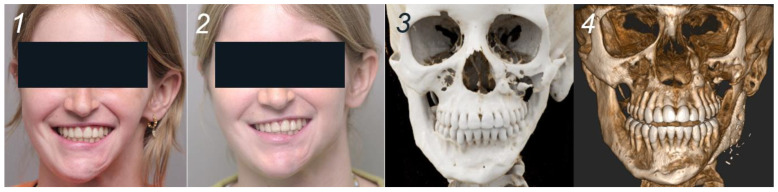
Clinical and radiological (CBCT) situation preoperatively and after 2.5 months, case 2. (**1**): Clinical situation pre-surgery—frontal plane. (**2**): Clinical situation 2.5 months post-surgery. (**3**): Radiological situation pre-surgery. (**4**): Radiological situation 2.5 months post-surgery.

In the second clinical case, the postoperative period showed beneficial healing of the PSI and donor site of the SCIAP flap. The aesthetic and functional results were satisfactory (Figure 5), although a discrete step was palpable, representing the transition of the mandible and the implant. No neurological deficits of the facial or trigeminal nerves were observed. CT imaging 2.5 months postoperatively showed a good position of the implant and absence of signs of infection around the screws (Figure 5).

Literature review showed one recent clinical case series of 13 patients using HA bioceramic PSIs similar to those used in our clinical cases [9]. These cases showed beneficial results in terms of biocompatibility, aesthetic outcomes, and osteointegration capacity [9]. Furthermore, mainly preclinical studies were available in the literature, describing benefits of bioceramics in comparison to conventional biomaterials in animal studies or in vitro. In this scoping review, the focus was on the comparison between HA implants, PEEK, autologous bone, and titanium implants.

Bioceramics can be divided into HA, tricalcium phosphate (α-TCP and β-TCP), bicalcium phosphate (BCP), and many more [12,13]. The Ca_10_(PO_4_)_6_(OH)_2_ composition occurs naturally in the mineral part of natural bone and accounts for approximately 50% of its weight [2]. Additionally, it is considered to be the least soluble bioceramic material [2,6,18]. It can promote bone growth without causing toxicity, inflammation, or undesirable immune reactions [6,7]. HA bioceramic implants function as osteoinductive and osteoconductive scaffolds and therefore imitate the technical characteristics of human bone after osseointegration [16,17,18,25] (Table 2). Removal of the implant is therefore not required.

The macrostructure of HA implants is important for the osseointegration process and a high level of porosity (>50%) is essential [9,18,25,32]. Although multiple designs have been proposed, a gyroid macrostructure showed the fastest bone neoformation [2,18,25]. The highly curved pore corners of the gyroid design enhance bone regeneration and cellular attachment [18,20]. Because of this structure, these HA PSIs have a low compressive strength of 1–12 MPa, in the range of human cancellous bone [2,25,26] (Table 2). However, the strength of the material increases when osteogenesis is obtained [6,25]. HA implants show no thermal conduction and have similar radiological properties compared to natural bone [9,12,16,17] (Table 2).

PEEK PSIs used in craniomaxillofacial reconstructive surgery show adequate strength, similar to human bone [15] (Table 2). However, their bio-inertia results in poor osteoconductive properties and difficult osteogenesis [7]. Occasionally, surface modification or HA coating is applied to the implant to enhance biocompatibility [7]. Slightly more infections were observed in PEEK PSIs [9,11,33] (Table 2). Furthermore, in vitro studies revealed that oral bacteria are more prone to adhere to the surface of a PEEK implant compared to titanium or HA [33] (Table 2).

Titanium PSIs exhibit good strength, biocompatibility, and resistance to infections [5,30] (Table 2). 3D-printed titanium PSIs are widely used in orbital floor repair, temporomandibular joint (TMJ) arthroplasty, and reconstruction of cranial and maxillofacial bone defects [5,12,34]. Disadvantages of titanium include the possibility of allergic reactions, thermal sensitivity, limited intraoperative adjustability, non-osseous behavior, and artefacts on CT and magnetic resonance imaging (MRI) [33,35] (Table 2).

Autologous bone grafting with non-vascularized or free vascularized bone flaps remains the gold standard in craniomaxillofacial reconstructive surgery [10,12,36]. It is widely used, and free vascularized bone flaps of the scapula, fibula, and iliac crest grafts provide high success rates in the current literature [36,37]. Intraoperative adaptations can easily be made, and immediate dental implant placement and prosthodontic rehabilitation is possible [38] (Table 2). However, there is significant donor site morbidity, infection risk, and possibility of graft resorption, especially in non-vascularized bone grafts [12] (Table 2).

## 4. Discussion

Driven by the desire to avoid bone grafting and reduce operative time, bioceramic HA blocks and particles were already used in craniomaxillofacial surgery in the 1980s [39,40]. However, it was difficult to prevent these particles from migrating [39]. Currently, these bioceramic materials can be used to manufacture PSIs [18]. The HA bioceramic PSIs provide a volumetrically stable scaffold of biocompatible material to restore craniofacial bone defects [18]. A PSI, regardless of the material used, is superior to standardized implants in terms of fit accuracy, reduction in surgery time and infection risk, stability, and implant–bone contact [4,41,42]. In particular, when surgical navigation is used, accuracy is enhanced [42].

### 4.1. Biocompatibility

HA bioceramic PSIs are biomimetic and eliminate the need for bone grafts [17]. They are osteoconductive and the gyroid macroporosities have the potential to guide osteoblasts and facilitate osteogenesis and fibrovascular ingrowth in vitro [18]. In vivo, osseointegration could not be objectively evaluated on CT images after six months postoperatively in the two cases (Figure 3). However, in the first clinical case, optimal bone contact and signs of osteogenesis were observed between the PSI and the bone. This indicates a beneficial healing, fibrovascular ingrowth, and mineralization around the implant. The HA bioceramic PSI proved beneficial because of its use as an inlay instead of an onlay in case 1. This led to an excellent aesthetic result in this important anatomical region (Figure 3). Case 2 showed similar radiological signs of osteogenesis (Figure 5). To be able to radiologically observe obvious signs of osseointegration, a longer follow-up time of twelve months is required [9,16].

In terms of biocompatibility, HA is superior to PEEK and titanium [9,13,18] (Table 2). The biocompatibility remains the highest when using non-vascularized or free vascularized bone flaps. The HA bioceramic PSI has a structure similar to that of mineral bone, without donor site morbidity or donor site infection risk. The affinity of HA for certain osteogenic, antiresorptive molecules and growth factors contributes to the osseointegration process [13]. The current literature shows good resistance to infection in comparison with titanium and PEEK [9,12,43]. This might be explained by the fast vascular ingrowth which facilitates the immune response [25]. On the other hand, infection risk is not only determined by the properties of the material, so further research in this field is necessary.

### 4.2. Biomechanical Properties

In comparison to titanium and PEEK, the mechanical strength of an HA PSI is inferior. The PSI has an initial brittleness and similar compressive strength as cancellous bone ranging from 1–12 MPa [6,27] (Table 2). Due to its brittle structure, the HA bioceramic PSI is vulnerable to perioperative fractures during handling and fixation [9]. However, it is accepted that the compressive strength of these HA implants increases in the function of the osseointegration process, and the fractured parts integrate as well [6,9].

Currently, dental implant placement in the reconstructed area is only possible in free vascularized bone grafts [38]. In vitro studies have reported that HA bioceramic PSIs may be suitable for reconstructing bone defects that require implant placement, although further clinical research is required [6].

Instead of acting as an onlay as titanium and PEEK do, the HA PSI becomes integrated into the bone, with similar biomechanical characteristics as human bone [16]. In other words, in the case of a fracture, the PSI will also fracture. Especially in the presented clinical case 1, a fractured HA PSI can lead to less trauma to the eye than non-fracturing titanium orbital PSIs.

Another advantage of HA bioceramics, compared to titanium, is the similar radiological behavior to human bone, resulting in fewer artefacts on medical imaging [14] (Table 2, Figure 3). Moreover, the osseointegration process can be actively monitored with CT imaging during follow-up [16] (Figure 3). Compared to titanium, HA bioceramic PSIs do not develop allergic reactions or thermal discomfort [5,35] (Table 2).

This review and clinical cases showed the advantages of 3D-printed HA bioceramic PSIs in comparison to conventional materials. However, it is important to assess the feasibility of applying the PSI and to evaluate the limitations of different types of biomaterials on an individual basis.

### 4.3. Surgical Benefits

In comparison to titanium PSIs, HA bioceramic PSIs can be easily adjusted during surgery because of their porous structure (Table 2, Figure 2) [6,18]. In addition, these implants can reduce surgical time significantly compared to autologous bone grafting and free flap surgery, which often requires a second surgical team [9,44]. In addition, there is no donor site morbidity or donor site infection risk.

### 4.4. Cost

The cost of a CAD/CAM manufactured titanium PSI is generally comparable to that of an HA bioceramic PSI. However, costs are depending on the case, the national reimbursement policy, the manufacturing company, and the size and design of the PSI [16]. Although the cost of a PSI is generally higher than that of a traditional implant, the operation time and postoperative hospitalization days are reduced when a PSI is used [9]. It has been studied that material costs only account for 20%–30% of the total expenses [33]. An accurate price comparison between traditional implants, PSIs, and various materials necessitates a comprehensive perspective considering the aforementioned factors.

### 4.5. Limitations and Strengths

A focused search was conducted to analyze a specific subset of materials for bone reconstruction in craniomaxillofacial surgery. Furthermore, we did not perform a systematic review, given the lack of available clinical studies about this topic. This introduces the potential for selection and publication bias. Nevertheless, our search was conducted from a rigorous critical perspective, prioritizing the inclusion of the most relevant articles addressing our clinical question.

A significant strength of this review is the inclusion of two successful clinical cases, each accompanied by detailed descriptions of digital planning, surgical procedures, and follow-up assessments supported by multiple clinical images. Our follow-up period was extended to at least six months, which is generally considered sufficient for assessing postoperative outcomes. However, longer follow-up periods are necessary to fully visualize the osseointegration of these implants on radiological imaging.

Furthermore, as only two cases were available at the time of this review, both cases were included regardless of their outcomes. This approach ensured the absence of selection bias. Notably, the use of bioceramic 3D-printed patient-specific implants is still emerging and not yet a standard practice.

### 4.6. Future Opportunities

3D printing can become the standard of care in transplantation medicine [19]. Despite surgical interest, clinical studies with long-term follow-up are currently limited. Additional in vitro research enhancing the biomechanical properties of the inherently brittle structures of HA could provide significant value to current knowledge. Introducing these novel techniques in clinical settings and gaining experience in their application can enhance our understanding of this material. Moreover, there is a growing interest in investigating the use of various grafts combined with growth factors.

## 5. Conclusions

3D-printed HA bioceramic PSIs have a great potential for reconstructive surgery in the craniomaxillofacial region. Literature review shows many advantages of these new implants in comparison to conventional techniques in terms of biocompatibility and biomechanical behaviour. Various applications are possible, as illustrated by the two cases presented in this paper. A longer follow-up period is necessary to radiologically evaluate the osseointegration process. The major challenge is the initial brittleness at time of implantation. Therefore, HA bioceramic PSIs are not yet favorable in the reconstruction of load-bearing anatomical structures. However, given their excellent biocompatibility and osseointegration capacity, we recommend their use in load-sharing anatomical structures for reconstructive or aesthetic purposes. Further research is required to evaluate the long-term effects of this promising biomaterial.

## Figures and Tables

**Table 1:** Two cases of reconstructive surgery using a bioceramic PSI in the craniomaxillofacial region, University Hospitals Leuven, Belgium.

Sex	Age (Years)	Etiology	Location	Approach	Bony Defect	Complications	Aesthetics	Follow-Up (Months)
Female	42	Intraosseous Hemangioma	Right supra-orbital rim	Hemicoronal	Supra-orbital and lateral orbital rim	None	Very good	6
Female	19	Parry–Romberg	Left mandibular angle	Extra-orally peri-angular	Atrophy of mandibular angle	None	Very good	6

**Table 2:** Characteristics of the most-used biomaterials for craniomaxillofacial reconstructions. Overview based on the international literature.

Outcome	HA Implant	PEEK	Autologous Bone	Printed Titanium	References
**Strength (MPa)**	1–12 at time of surgery 30 after 9 weeks	140–170	Cortical bone: 100–230 Cancellous bone: 0.22–10.44	190–639 (2 mm printed plate)	[5,6,7,10,26,27,28,29]
**Osteoinductivity**	Good, very good in combination with BMP2	/	Excellent	/	[6,7,10]
**Osteoconductivity**	Very good	Poor	Excellent	Poor	[1,11,12,30]
**Osseointegration**	Very good	Poor	Excellent	Very good	[1,11,12,14,31]
**Infection risk**	Less than PEEK and titanium	Greater infection risk Prone to bacterial adhesion	Donor site morbidity	Less prone to bacterial adhesion	[9,11,15,16]
**Thermal behaviour**	No thermal sensitivity Thermostable	No thermal sensitivity Thermostable	No thermal sensitivity Thermostable	Thermal sensitivity Thermostable	[1,11]
**Volumetric stability**	Volumetrically stable	Volumetrically stable	Possible resorption	Volumetrically stable	[1,11]
**Radiologic** **behavior**	Similar to bone	Radiolucent	Similar to bone, artefacts of osteosynthesis screws	Artefacts on MRI and CT	[12,15,17,18]
**Intraoperative** **adjustability**	Possible	Possible	Good	Limited	[11,17,20]
**Main advantage**	Mimics cancellous bone	Strength	Biocompatibility	Strength and biocompatibility	[11,12,21,23]
**Main disadvantage**	Low strength	Lack of osseointegration	Resorption rate and Donor morbidity	Thermal sensitivity	[11,12,14,25,32]
**Conclusion**	Poor strength, good osteoinductivity/conductivity and osseointegration. Good thermal and radiological behavior, good volumetric stability. Possible to adapt intraoperatively.	Strength similar to bone, poor osteoconductivity and osteointegration. Prone to bacterial adhesion. Good thermal and radiological behavior, good volumetric stability. Possible to adapt intraoperatively.	Good strength for load-bearing structures. Excellent osteoinductivity, osteoconductivity, and osseointegration. Good thermal and radiological behavior, poor volumetric stability. Possible to adapt intraoperatively.	Excellent strength for load-bearing structures. Poor osteoconductivity and osteointegration. Suboptimal thermal and radiological behavior. Good volumetric stability, and difficult to adapt intraoperatively.	

## Data Availability

The authors confirm that the data supporting the findings of this study are available within the article.

## Abbreviations

3D	Three-dimensional
α-TCP and β-TCP	α- and β-tricalcium phosphate
BCP	Bicalcium phosphate
BMP2	Bone morphogenic protein 2
CAD/CAM	Computer-aided design and manufacturing
CBCT imaging	Cone-beam CT imaging
CT scan	Computed Tomography scan
FGF	Fibroblast growth factor
HA	Hydroxy apatite
MPa	Mega Pascal
MRI	Magnetic resonance imaging
PET-CT scan	Positron Emission Tomography CT scan
PICOS criteria	Patient population, intervention, comparison, Outcome, and study design
PEEK	Poly-ether-ether-ketone
PSI	Patient-specific implant
QoL	Quality of life
SCIAP flap	Superficial Circumflex Iliac Artery Perforator flap
TMJ	Temporomandibular joint
TPMS	Triply periodic minimal surface
VEGF	Vascular endothelial growth factor
